# The Role of Junior Adolescents’ School Well-Being/Ill-Being Characteristics in School Anxiety Variations

**DOI:** 10.3390/ejihpe11030065

**Published:** 2021-08-17

**Authors:** Rail M. Shamionov, Marina V. Grigoryeva, Aleksey V. Sozonnik, Elena S. Grinina

**Affiliations:** Pedagogical and Special Education, Faculty of Psychological, Saratov State University, Astrakhanskaya St., 83, 410012 Saratov, Russia; grigoryevamv@mail.ru (M.V.G.); elena-grinina@yandex.ru (E.S.G.); sznnik@mail.ru (A.V.S.)

**Keywords:** adolescent, school anxiety, school wellbeing, academic adaptation, factors

## Abstract

Difficulties that junior adolescents (aged 11–13 years old) experience in terms of academic adaptation, which are indicated through school anxiety and academic wellbeing characteristics, often lead to a dramatic decrease in academic performance, behavioral problems, and deterioration of their health. The purpose of this investigation is to determine the structure of characteristics of school wellbeing/ill-being of junior adolescents and their role in variations of school anxiety, which largely define academic adaptation. In this study, based on positive psychology and a systematic approach, the level of distinctiveness of characteristics of school wellbeing is carried out with the help of comparative analysis; the factor structure of these characteristics is identified; the characteristics of wellbeing and their coordinated combinations (factors) are determined as predictors of school anxiety. The sample consisted of 120 students of the 5th–7th grades, aged M = 11.5; SD = 1.04 (49.2% girls, 50.8% boys) who attended Saratov secondary schools. To study the level of school anxiety, we used the Philips’ School Anxiety Scale (SAS), and indicators of school wellbeing were measured with the original scales developed by the authors of the study. Statistical processing of the results was carried out with regression analysis and factor analysis. The results showed that the school wellbeing of junior adolescents forms a complex structure that includes cognitive, personal, emotional, social, and psychophysiological characteristics of school life. It was found that from 16% up to 53% of the deviation of variables characterizing school anxiety is conditioned by the assessment of variables characterizing emotional states, the ability of self-regulation, cognitive capabilities, and interest in learning. The study determined a high level of tension in adolescents in the field of emotions’ self-regulation, unpleasant physical sensations at school, before and after attending school, in the course of planning their school day and reflecting on educational activities. The most powerful factors of school anxiety in junior adolescents are physical distress, low ability to self-regulate and social adaptation, lack of independence in a learning activity, and personal immaturity.

## 1. Introduction

The problem of adolescents’ school wellbeing is of genuine concern, despite the seemingly well-thought issues related to organizing their adaptation to the academic environment, the system of interrelations between all of its subjects, and the educational process. Adolescents face a variety of problems that lead to anxiety and frustration. These include lack of attention to individual characteristics of their development [1]; school responsibilities and excessive academic demands; intense relations with both peers and adults [2]. Modern psychological research, which is carried out worldwide, has recorded the highest rates of school anxiety in children of this age category, specifically in Russia. An increased level of school anxiety is a trigger for a number of somatic disorders. These include: endocrine system disorders, eating disorders, metabolic disorders, immune disorders, nervous system disorders [3]. Apart from somatic diseases, psychological disorders also occur in children as anxiety levels increase. This fact was confirmed by the studies of European psychologists [4].

The optimal level of schoolchildren’s anxiety assures their academic and communicative success and promotes their general wellbeing [5,6]. The psychological wellbeing of a schoolchild is a marker of good psychological functioning. According to most researchers, moderate anxiety has a positive effect on development, as it acts as adaptive anxiety that contributes to a person’s success. Moderate anxiety is the marker of relative emotional wellbeing. On the contrary, excessive calmness is a sign of distress and presence of psychological problems, expressed through inappropriate behavior and latent aggression; in some cases, it may indicate depressive tendencies, etc. It has been proven that increased anxiety levels in schoolchildren are accompanied by other psychological disorders [7]. Pathological anxiety interferes with healthy functioning and the development of the system [8]. When anxiety symptoms remain unnoticed or are misinterpreted and viewed as behavioral and academic problems, children’s social, academic and emotional wellbeing can be under threat [9]. High levels of anxiety can lead to avoidant disorder in a student, which manifests through frequent truancy, refusal to attend school, avoidance of social interaction, decreased self-esteem, etc. Increased anxiety, which is expressed through frequent absenteeism, is a characteristic symptom of a student’s low level of school wellbeing. Excessive school anxiety indicators cause low self-esteem and a tendency to use self-deprecation strategies [10], which is expressed through the feeling of unhappiness, dissatisfaction with oneself and one’s life, which are the key aspects of personal wellbeing/ill-being. In this case, the question of how academic anxiety and school wellbeing of adolescents has correlated becomes significant for psychology.

When studying personal wellbeing in a particular environment, it is necessary to identify its components at a given time and its factors. Thus, Polivanova [11] views schoolchildren’s wellbeing as a complex of “individual” components: psychological wellbeing (the idea of oneself and one’s life); cognitive wellbeing (knowledge and skills); social wellbeing (quality of social communication); physical wellbeing (health) and material wellbeing (financial capabilities of the family). The adolescent’s academic wellbeing model consists of several factors that affect their anxiety level, i.e., school conditions, social relations, self-realization tools, and health. The latter factor refers to physical health and wellbeing, which is viewed as the satisfaction of basic needs, i.e., to have, to love, and to be [12]; satisfaction; happiness; the high quality of life. According to PISA-2018 data, schoolchildren characterize wellbeing at school as satisfaction with their appearance, relations with parents, and school events [13].

Indicators of adolescents’ subjective wellbeing are their wellbeing at school; good relations with teachers; interest in academic tasks; educational motivation; responsibility for homework; attentiveness in the classroom, a positive academic image of themselves; social integration in the classroom. In addition to all other factors, social relationships, mainly with peers, affect schoolchildren’s academic wellbeing to a greater extent [14]. They influence how a child will develop interpersonal relationships in the future [15,16], the realization of his/her personal potential, as well as mental health [17].

An adolescent’s wellbeing depends on how comfortable, meaningful, and enjoyable his/her life is. Wellbeing at school consists of a complex of external and internal components, i.e., positive emotions related to school as a whole, positive academic self-concept, deriving pleasure from school activities, an optimal level of academic anxiety, absence of somatic disorders associated with educational activities, and absence of social problems in the educational environment [18].

It should be noted that adolescents’ wellbeing is often viewed through the prism of their academic success, but little attention is paid to whether a child feels comfortable with this. The modern education system aims to increase educational achievements, which serve as a guarantee of personal subjective wellbeing in adult life. Analysts who survey school life note that the quality of life of an adolescent, satisfaction with it at a given point in time should not be sacrificed for future success and achievements [19]. Despite the fact that an adolescent’s wellbeing is an assisting factor in overcoming anxiety, which contributes to the development of “immunity” to stressful situations, it actualizes their potential; it also serves as an indicator of an adolescent’s academic adaptation. The academic wellbeing of a teenager is viewed as a system of experiences reflecting his/her idea of their place within the “environment-child” system. Their state depends on how intense these experiences are [20]. The sign of a student’s school wellbeing is positive mood, orientation towards the future, success, satisfaction with studies, general self-esteem and private self-esteem (appearance, success in any type of activity), social orientation, decision-making ability, good sleep, energy, appetite and optimal anxiety level [21].

Along with studying the subjective wellbeing of schoolchildren, school anxiety is being studied quite widely as well. Children and adolescents who have school anxiety are apt to show poor academic performance, symptoms of acute anxiety, tension or disturbance, depression, melancholy, ambivalent and hyperactive behavior, and a distorted self-image [22]. It is possible to identify some structural components of school anxiety-like social and general anxiety [23]; anticipatory anxiety; anxiety connected to school performance, and generalized anxiety disorder [24]. Academic anxiety deserves special attention concerning some of its aspects like mathematical [25,26,27] and test anxiety which appears to be the most studied [28,29].

Studying the correlation between subjective wellbeing and anxiety indicates that there is a steady connection between them [30,31,32]. Studies of schoolchildren in this context are pretty fragmental.

Derdikman-Eiron et al. [33] studied the subjective wellbeing of adolescents with symptoms of anxiety and depression. It was found that symptoms of depression and anxiety characterize 10.2% of students aged 13–19 years, and they are significantly more common in girls than in boys. The study of the correlation between anxiety and subjective wellbeing indicated that boys without symptoms of anxiety are characterized by a higher level of subjective wellbeing than girls. In contrast, the correlation between anxiety and subjective wellbeing regarding gender differences in anxious adolescents was not detected. As it was noted later on by Moksnes et al. [34], anxiety and subjective wellbeing in adolescents may change during the school year. Thus, girls had higher rates of anxiety than boys at the beginning of the year, while boys had significantly higher rates of mental wellbeing than girls. Still, at the end of the year, the increase of anxiety and the decrease of wellbeing were indicated both in boys and girls. Holopainen et al. [35] draw our attention to the fact that those students who experienced learning difficulties had a lower level of subjective wellbeing.

It is noteworthy that most research studies the relationship between school wellbeing and one specific type of anxiety—test anxiety [36,37]. The study of Putwain et al. [38] reveals a negative relationship between school wellbeing and anxiety while testing. According to the study results of Kaplan [39], the connection between subjective wellbeing and anxiety is low and middle-rated. It is also noted that students with a higher level of subjective wellbeing had lower anxiety and general emotional problems. Steinmayr et al. [40] concluded that test anxiety negatively impacts cognitive and affective components of life satisfaction in students of the 11th grade. Attention is also paid to the prevention and correction of school anxiety and ill-being with the help of various technologies and practices, through sports [41], group interventions [42], cognitive-behavioral interventions [43], etc.

Thus, current studies present separate attempts to study the relationship between subjective wellbeing and anxiety in schoolchildren. However, there is still no research that provides a system and structure to the issue.

Based on the foregoing, it can be assumed that anxiety level directly correlates with the student’s wellbeing level. However, it is important to establish a connection and identify those characteristics of school wellbeing/ill-being that significantly contribute to variations in school anxiety and should be viewed as the most important objects (targets) of psychological intervention.

## 2. Purpose and Hypotheses

### 2.1. Purpose

The purpose of this research is to determine the structure of characteristics of school wellbeing/ill-being in adolescents and their role in variations of school anxiety determining academic adaptation in general.

### 2.2. Hypotheses

**Hypothesis** **1 (H1).** *The school wellbeing of junior adolescents forms a complex structure that includes cognitive, personal, emotional, social, and psychophysiological characteristics of school life. Previous studies have shown that the ability of a teenager to cope with emotional problems plays an important role in school wellbeing [44], as well as the ability to set up relationships with other people [14], sufficient development of cognitive functions (memory, attention, thinking) [45]. Thus, this study assumes that the more these characteristics are developed, the higher rate of school wellbeing can be observed*.

**Hypothesis** **2 (H2).** *Characteristics of school ill-being primarily determine school anxiety. School anxiety as an integrated feeling of emotional discomfort, anticipation of negative events as well as fear is associated with particular events of school life—the situation of external assessment [28], relationships with peers [23] and relationships with teachers [29], and ability to cope with academic tasks [46] and with other characteristics, representing school wellbeing. Based on the discussion above, this study suggests a high level of determination of school anxiety (as an integral assessment of discomfort) by individual characteristics of school wellbeing*.

**Hypothesis** **3 (H3).** *The strongest predictors of school anxiety are the enlarged groups of characteristics of school wellbeing, reflecting negative values of self-respect, desire for development, and physical state, self-regulation, and social adaptation. Previous studies have shown the ambiguity of school anxiety causes [1,4]. It can be associated with the possibility to combine different characteristics of school wellbeing/ill-being that explain variations of anxiety. Therefore, the insertion of (independent) variables reflecting groups of characteristics of school wellbeing obtained from factor analysis into the regression equation will allow us to identify the most significant combinations for experiencing anxiety*.

## 3. Materials and Methods

### 3.1. Study Design

The logic of the study is as follows. To test the first hypothesis, we analyze the arithmetic means and factor structures of school wellbeing. The obtained factors are viewed as related ones according to the principle of joint variability. The data obtained from factor analysis are interpreted as types of school wellbeing or ill-being, which are indicated on the basis of related characteristics. To test the second hypothesis, we study the regression analysis results, where school anxiety indicators are the independent variables, and indicators of school adolescents’ wellbeing/ill-being are dependent variables. Then, to test the third hypothesis, a regression analysis is performed. The dependent variable is the general indicator of school anxiety, and the independent variables are new enlarged variables obtained from factor analysis of school wellbeing/ill-being characteristics.

### 3.2. Participants

The study involved 120 junior adolescents of the 5th-7th grades, 10–13 years (mean (M) = 11.5, SD = 1.04 years) (*n* = 59 (49.2%) girls, *n* = 61 (50.8%) boys). The study involved students of secondary schools: 5-graders—40, 6-graders—40, 7-graders—40 (2 classes each, the average number of students in each class is 20 people). The sample was consistent with the population of junior adolescents studying at city secondary schools in age, gender, education, socio-demographic characteristics.

### 3.3. Measurements

Junior adolescents’ school wellbeing was defined with the unique scales that were developed by the authors of the study based on the choice of one of the four answer options (“yes, almost always,” “not always,” “sometimes,” “no”).

In the scales’ development, the authors use the approach which links wellbeing with psychological adaptation indicators. On the one hand, personal subjective wellbeing is an important outcome and indicator of psychological adaptation [47,48,49,50]. On the other hand, personal wellbeing is a condition for further psychological adaptation under new conditions of activity [45,51,52].

In addition, earlier studies, which developed the concept of active school adaptation, found that its main components were: cognitive characteristics that contribute to effective mastering of academic skills and self-organization of a student in the educational process; emotional wellbeing and positive intrinsic motivation for learning [53]. The assessment of each scale for compliance with the measured phenomenon was carried out by 5 researchers dealing with the problem of school wellbeing, who were asked to evaluate each scale from 0 to 4 for applicability. Afterward, there was a discussion of each scale formulization and tool improvement. The average acceptance score for each scale ranged from 3.6 to 4. All scales found a significant positive correlation with student satisfaction with school life, measured by direct ranking using the Likert scale.

Examples of scales: “Are you usually in a good mood at school?”, “Are you comfortable with communication with teachers?”, “Do you quickly understand the teacher’s explanations?” etc. The scales developed by the authors of the article were proposed to 5 experts working in the field of developmental psychology, educational psychology, who assessed them on a 5-point scale in terms of their compliance with various aspects of school wellbeing. Next, we selected questions that received more than 4 points on average. All scales were checked for consistency using the Cronbach Alpha method and gave acceptable results (α = 0.81–0.86).

To define characteristics of school anxiety as one of the aspects of adolescents’ adaptation to school, we used Philips’ School Anxiety Scale (SAS). The scale includes 58 questions, which have to be answered with a “yes” or a “no.” This method’s application allows diagnosing the general level of school anxiety, as well as its individual aspects: experiencing social stress, frustration related to the need to achieve success, fear of self-expression, fear of the situation when one’s knowledge is tested, fear of not meeting expectations of others, low physiological resistance to stress, problems, and fears regarding relations with teachers. The scales showed a good level of internal consistency (Cronbach’s Alpha test showed acceptable results α = 0.76–0.84).

### 3.4. Procedure

The empirical data collection was carried out in the first half of the 2020–2021 academic year. The survey had been conducted with the permission of the school administration and parents of those adolescents who participated in the study. The method of randomized selection was used. Classes of secondary schools were selected randomly. The selection of classes was not previously made, which ensured the representativeness of the sample.

The data sources used in this study were accurate and reliable. While developing the tool, we used data gained from focus groups of school psychologists (5 people) and parents of students (three focus groups of 7 people each) concerning school problems and the school wellbeing of junior adolescents. The participants of the focus groups were asked to name the signs of school wellbeing and ill-being of 5–7 graders. The number of focus groups was dictated by the necessity to check and recheck the criteria for assessing the school wellbeing in adolescents by parents of students and school psychologists. The data gathered from school psychologists and parents were grouped by modality into cognitive, personal, emotional, and social characteristics. Then, each item of the questionnaire was assessed by the 5 psychologists dealing with problems of school wellbeing for compliance with the phenomenon of school wellbeing in adolescents. The final version of the questionnaire included those characteristics of school wellbeing or ill-being in adolescents that appeared to be common for all participants of focus groups, sporadic ones were not included in the questionnaire. Signs of school wellbeing and signs of ill-being were included in each group of characteristics reflecting cognitive, personal, emotional, and social indications of school wellbeing. For a more accurate determination of psychophysiological indications, the group of psychophysiological signs included symptoms of negative psychophysiological state (headache, abdominal discomfort, back/arms/legs, etc., muscle fatigue). The criteria for the school wellbeing of adolescents obtained as a result of discussions in focus groups correlate with previous studies [13,14,18], which indicates the content validity of these criteria. The studies were carried out in two secondary schools with no specific parameters for selection (without a profile), thereby ensuring confidence that the sample will represent the target population of junior adolescents of secondary school. The survey of students was conducted in the presence of a school psychologist, providing psychological assistance to students if necessary.

All parents of the children provided their informed consent for inclusion before they participated in the study. The experimental studies were performed in accordance with the Ethical Standards (2000) and were approved by the local Research Ethics Committee (Protocol No. 2 of August 20, 2020) of the Saratov State University (Faculty of Psychological, Pedagogical, and Special education). The study was conducted at the beginning of the academic year for 2 months.

### 3.5. Statistical Analysis

We used the statistical software package IBM SPSS Statistics + PS IMAGO PRO to process the primary data.

First, the scales were checked for internal consistency using the Cronbach’s alpha coefficient, and the data were checked for the normality of the distribution using the Kolmogorov–Smirnov’s test and the analysis of asymmetry and kurtosis indicators. All tests showed an acceptable result of matching the distribution to the normal one. Then the socio-demographic data were studied using descriptive statistics (displayed in averages, standard deviations, and percentages). After that, a factor analysis (EFA) of indicators of school wellbeing/ill-being was carried out using the main components method and confirmatory factor analysis (CFA) with a 1 and 4-factor solution. Then a regression analysis was carried out. Independent variables were indicators of wellbeing (at the beginning separately, then new variables based on factor analysis), and dependent variables are indicators of school anxiety.

## 4. Results

### 4.1. The Structure of School Well-Being

Comparison of the indicators of mean and standard deviations of the characteristics of school wellbeing/ill-being (see Table 1 below) revealed a relatively high level of tension in schoolchildren in the field of self-regulation of situational emotional disorders, unpleasant physical sensations at school, before and after attending school, and planning their school day. Reflection on classwork, cognitive motivation, assessment of cognitive development, self-assessment of academic success, and regulation of states using special techniques and means turned out to be quite tense. Obtained data demonstrated a very high level of differentiation within the sample for these parameters: standard deviation values indicate a strong scatter of indicators. The highest indicators were satisfaction with communication with peers, teachers, relations with teachers, taking care of school supplies, where adolescents expressed the desire to be a good student, and a positive attitude towards educational material was observed.

The value of Kaiser–Meyer–Olkin measure of sampling adequacy demonstrated a high rate of sampling adequacy for factor analysis. Bartlett’s test of sphericity indicated a statistically reliable result (*p* < 0.01); the correlations between the variables differ from 0 significantly. The Cronbach’s Alpha indicators show an acceptable level of consistency of the obtained new variables based on factor analysis. In addition, the indicators of Kolmogorov–Smirnov’s test showed that there were no significant differences from the normal distribution.

The confirmatory factor analysis (CFA) confirmed a qualitative match with the results of the exploratory analysis (EFA). Comparison of the results with an alternative model to the 1 factor (Table 2) helps to opt for a 4-factor solution. Factor analysis allowed to single out four factors, which involved various indicators of school wellbeing. Based on these factors, we offered an interpretation of the four types of school wellbeing for the 5th–7th-grade students. The first factor included indicators that characterize: reflection on classwork and mistakes that were made, positive attitude to academic material, a communicative indicator for assessing relations with teachers, emotional indicator characterizing satisfaction with these relations, self-regulatory indicator, which is marking their autonomy in the process of using school supplies and taking care of them; two motivational indicators characterizing cognitive motivation and motivation for achieving success; a personal indicator of the desire to actualize this motivation.

Indicators of school wellbeing listed above mainly characterized the attitude towards acquired knowledge as a value and main goal of school life; attitude towards a teacher as an important person who is sharing knowledge; the need for self-organization in the learning process and striving for success. This factor could be defined as “school wellbeing based on the desire for knowledge and self-organization.”

The second factor combined the following indicators: emotional—satisfaction with studies; communicative—ability to defend one’s point of view; regulatory—ability to organize one’s schoolwork at home; personal—the autonomy of academic work and self-assessment of school success. We defined this factor as “school wellbeing based on autonomy and personal maturity.”

The third factor included indicators of satisfaction with relations with classmates; well-developed cognitive sphere (attention, memory, comprehension, speech); lack of unpleasant physical sensations before school, at school, and after it; self-regulation of mental states; self-assessment of adaptability. The third factor could be defined as “school wellbeing based on physical wellbeing, self-regulation, and social adaptation.”

The fourth factor united two communicative indicators (the desire to be better in the eyes of others, ability to fight back an offender), regulatory indicator (using self-regulation methods), and the personal indicator (planning a school day). This factor was defined as “school wellbeing based on self-respect and desire to develop.”

### 4.2. Characteristics of School Well-Being as Predictors of School Anxiety

As we can see from Table 3, social stress is most determined in terms of school ill-being indicators. Social stress is significantly reduced if adolescents are in a good mood at school (β = −0.18); they are satisfied with their relations with classmates (β = −0.31); can defend their position in a dispute (β = −0.31); use methods and tools that improve their physical wellbeing, increase their self-confidence at school (phone, social networks, believing in superstitions, self-immersion, avoidance of communication, talks with friends, etc.) (β = −0.24). These predictors are fairly foreseeable for reducing social stress since they are associated with communicative characteristics accompanying social interactions with peers and integral indicators of mood and self-regulation skills. One of the predictors of social stress decrease indicated the absence of external educational motivation for avoiding trouble and parents’ negative reaction to their grades at school (β = −0.24) (see Table 3), which, in turn, indicates the possibility of schoolchildren’s social wellbeing in the absence of this assessment. Table 3 shows that an increase in students’ social stress at school is determined by autonomy to do their homework and organize their studies (β = 0.19), which can be possibly explained by lower assessment of the results of their autonomous study and stress related to social contacts with the adult participants of educational interaction. The last two predictors of a decrease in students’ social stress (lack of external academic motivation for avoiding trouble, negative assessment from parents, and autonomy in completing homework and organizing their studies) showed a certain contradictory link between schoolchildren’s autonomy in their studies and the autonomy results’ assessment by parents.

Frustration/barrier on the way to success at school decreases due to lack of negative emotions (β = −0.24), the prevalence of good mood (β = −0.24), effective functioning of cognitive processes (β = −0.19) and high level of self-assessment in terms of finding it easy to study at school (β = −0.2) (Table 3). We can see that academic success was primarily determined by positive emotional background and good cognitive development.

Fear of self-expression at school was significantly reduced in adolescents due to the action of the following predictors: absence of manifestations of negative emotions (sadness, resentment, anger) (β = −0.51) and absence of external educational motivation for avoiding trouble (β = −0.24) (Table 3). Therefore, expressive components of behavior at school, ease of expressing one’s opinion, absence of serious communication barriers were primarily associated with the lack of focusing student’s attention on the negative aspects of school life. Fear of self-expressing was increased by the cognitive predictor “emotionally-positive attitude to lesson material” (β = 0.29) and self-regulatory predictor “using methods and tools that improve one’s general state and self-confidence at school” (β = 0.2) (Table 3). As we could see, these predictors were to a greater extent associated with internal cognitive and regulatory processes. Perhaps, the fact that a student turned to the phenomena of his/her inner world, i.e., that he/she is getting pleasure from learning new material, is able to identify his/her state and has good psychophysiological regulation, do not actualize and do not form free acts of self-expression.

The fear of knowledge check decreased in adolescents in the absence of negative emotions (sadness, resentment, anger) (β = −0.29) and the presence of the skill to calm down quickly if one is upset (β = −0.26) (Table 3). We observed that the absence of negative emotional response and ease of regulation of negative emotions decreased fear of testing how much adolescents know. This can be obviously explained by the decrease in general anxiety at school; when having a positive or neutral emotional background, a teenager does not focus on the expectation of trouble, including trouble caused by testing their knowledge. Table 2 shows that planning a school day increases a student’s fear related to testing his/her knowledge (β = 0.41). This was possibly happening due to the adolescent’s inclusion of knowledge check situations in his/her plans, thinking through and reflecting on what can be shown at the stage of knowledge check and how this can be done. It could also be related to the awareness of some shortcomings in the results of their studies, which can lead to an increase in anxiety and fear.

Predictors of a decrease in fear of not meeting somebody’s expectations were the absence of negative emotions (β = −0.36), well-developed cognitive sphere and ease of learning (β = −0.24), autonomous learning (β = −0.21), and the lack of extrinsic motivation for avoidance of trouble (β = −0.22) (Table 3). The above-listed indicators of school wellbeing obviously provide opportunities for a student to meet the expectations of teachers and parents to a higher degree.

An increase in resistance to school stress was associated with the adolescents’ ability to quickly calm down if they are upset about something (β = −0.28) (Table 3), which implied high practical importance of developmental and corrective psychological work with school students during which they can develop emotional self-regulation skills.

Fear of a teacher decreased due to absence of negative emotional background (β = −0.24) and good emotional self-regulation skills (β = −0.4) and increased when there was high satisfaction with relations with classmates (β = 0.26) (Table 3). The mechanism of the first two predictors of fear of a teacher was clear. It was associated with a favorable emotional state of adolescents and a quick return to this state in situations of tension or conflict in the course of interaction with a teacher. The latter predictor most likely indicates that there were different mechanisms of social wellbeing in situations of communication with classmates and situations of communication with teachers. It is also possible that adolescents, due to the actualization of the need for communication with peers, cannot equally harmoniously perceive themselves in communication situations with classmates and teachers.

In general, the decrease in school anxiety was determined by the absence of negative emotions (β = −0.26), good mood (β = −0.22), ease of emotional self-regulation (β = −0.32), while its increase was associated with mental modeling of the answer in class (β = 0.28) (see Table 3). The most functional predictors of a decrease in school anxiety were the absence of negative emotions (sadness, resentment, anger), ease of emotional self-regulation, and absence of external educational motivation to avoid trouble (see Table 3).

The result of the regression analysis is a model (Table 4) in which (1) school wellbeing is based on physical wellbeing, self-regulation, and social adaptation and (2) school wellbeing based on autonomy and personal maturity explained 21% of the total variance of school anxiety.

## 5. Discussion

Comparative analysis of indicators of school wellbeing made it possible to identify the most and least manifested characteristics. A high level of tension in adolescents was established in areas of school life that characterize the system of emotional self-regulation, self-control, and organization-related function of activity. Tension in the field of cognitive activity and academic success is somewhat less pronounced. However, according to the study results of Moksnes et al. [34], the stress associated with school performance has a significant negative impact on life satisfaction. In contrast, the stress experienced from communication with a teacher is extra significant. In areas of school life that characterize the system of emotional self-regulation, self-control, and organization-related function of activity, we observed very high differentiation of indicators, which means that some adolescents have higher indicators while others have lower indicators of these characteristics. These areas are obviously the most important ones for school adaptation [46]. On average, characteristics of school wellbeing are most pronounced within the system of relations (with teachers and peers), attitudes towards academic work. These findings are consistent with research findings [54,55] that identify social factors of adolescents’ school wellbeing and the importance of emotional self-regulation factors [44]. Intrinsic motivation for academic work, lack of fear of external evaluation and negative consequences also play a significant role in school adaptation [56]. The collected data correspond to the results of other empirical studies [34], expert assessments (made by professional psychologists), and assessments of students’ parents, according to which junior adolescents are more stressed about their abilities to organize their activities and academic performance.

Factor analysis allowed us to identify four factors that characterize different aspects of the school wellbeing of adolescents. They are: striving for knowledge and self-organization (the most powerful factor); autonomy and personal maturity; good physical state, self-regulation, and social adaptation; self-respect and desire to develop. This sequence characterizes the value-related sequence, which determines the importance of various components of school wellbeing. These data contribute to debunking the myth that the main thing related to adolescent’s school wellbeing is the system of relations with peers and teachers. To a large extent, these relationships are important in primary school age because they influence the optimism, school motivation, creativity, satisfaction with the school life of students [57]. However, cognitive activity and its motivators are still first, while relations-related factors take second place. The success in cognitive activity as a significant factor of school wellbeing of adolescents was noted earlier as well [46]. At the same time, relations-related factors were identified in several studies as the primary [58]. Finally, the most important circumstance of the school wellbeing in junior adolescents is a developed (in accordance with age) system of self-regulation and self-control, which are the resources of their psychological wellbeing at the following stages of education [59]. The psychological wellbeing of schoolchildren is largely determined by a high level of school wellbeing and a low level of school anxiety [60].

As a result of regression analysis, it was found that 16% to 53% of the deviation variables characterizing school anxiety are conditioned by evaluation of various characteristics of school wellbeing/ill-being of junior adolescents. The most important ones are variables characterizing emotional states, ability to self-regulate, cognitive capabilities, and interest in learning. From the obtained data, it follows that about half of the variations of social stress, frustration/barrier on the way to success, and fear of adolescents not meeting expectations are explained by the characteristics of school wellbeing. The strongest predictors are manifestations of negative emotions, subjectively poor cognitive development, and motivation to avoid trouble. Since school anxiety and characteristics of ill-being are the most obvious signs of school maladjustment [53,61], it can be assumed that tension in understanding academic material, subjective deficiencies in cognitive functions (memory, attention, thinking), lack of internal motivation due to this, and problems of emotional self-regulation are its most important causes.

These data indicate that social stress, frustration, and fear of not living up to somebody’s expectations are not caused by social tension proper, but rather by the internal problems of adolescents, in particular their self-esteem and self-respect [34]. This is consistent with the data of researchers who note the high importance of social factors in the wellbeing of schoolchildren (parents’ expectations, ways of motivating teenagers, etc.) [54,62] and evidence that classroom climate is loosely associated with social-emotional stress [63].

Finally, as a result of the regression analysis, it was found that two new variables of school wellbeing based on physical wellbeing, self-regulation and social adaptation, and school wellbeing based on autonomy and personal maturity explain nearly 21% of variations of adolescents’ school anxiety. It is obvious that the combination of indicators like satisfaction with peers’ interrelationship; sufficient development of cognitive sphere (attention, memory, comprehension, speech); the absence of unpleasant physical sensations before, at and after school; self-regulation of mental-state; self-assessment of adaptability and satisfaction with the learning activity, capability to defend one’s position in a dispute, capability to organize one’s learning activity at home, autonomy at learning activity and self-assessment of academic success is significant for reducing school anxiety. The results obtained from previous researches also support this assumption. In particular, school anxiety can be reduced by cognitive-behavioral interventions [64], in which the deactivation of the main aspects of anxiety allowed to reduce it slightly, but it turned out that there are some other factors of anxiety. At the same time, learning and social activity, perseverance, and persistence also prevent the development of school anxiety [7,14].

Many studies show that the characteristics of school wellbeing in adolescents constitute grounds for the prevention of school anxiety, their adaptation to the school environment [47,60], and psychological wellbeing in general [18,61]. The results of our research confirm the previously collected data on the significant role of junior adolescents’ school wellbeing/ill-being characteristics in school anxiety variations and contribute to the further development of the study in this realm. The value of the data collected by us lies in identifying specific characteristics of school wellbeing/ill-being, which are significant for predicting the symptoms of school anxiety. We have identified distinct characteristics and combinations, which were presented as factors and predictors of school anxiety. The results of our study open up the prospect for studying the substantive characteristics of psychological wellbeing not only in young but in senior adolescents as well and determine the scope of “individual” psychological assistance to them.

## 6. Conclusions

As a result of the study, the hypotheses put forward were confirmed. Junior adolescents’ school wellbeing forms a complex structure that includes school life’s cognitive, personal, emotional, social, and psychophysiological characteristics. The ability to emotional self-regulation in academic situations; signs of psychophysiological wellbeing before, at, and after school; tendency to plan a school day; reflection on classwork and learning motivation are indicated to a lesser degree within the structure of school wellbeing. Such values as satisfaction with the process of communication with peers and teachers and the process of taking care of school supplies have the highest score; adolescents showed a desire to be a good student, and a positive attitude to the educational material was expressed as well. Factor analysis identified four factors corresponding to four types of school wellbeing: “school wellbeing based on the desire for knowledge and self-organization,” “school wellbeing based on autonomy and personal maturity,” “school wellbeing based on physical wellbeing, self-regulation, and social adaptation,” and “school wellbeing based on self-respect and desire to develop.”

School anxiety is determined primarily by characteristics of school ill-being. Regression analysis showed that social stress is the most deterministic factor among other indicators of school ill-being, and its reduction is associated with a student’s good mood at school; satisfaction with relationships with classmates; the ability to defend one’s position in a dispute; the use of techniques and means that improve wellbeing and increase self-confidence at school (phone, social networks, belief in superstitions, self-immersion, avoidance of communication, talking with friends, etc.); lack of external educational motivation to avoid troubles and negative reaction of parents to a student’s grades. A student’s academic success is primarily determined by his/her positive emotional state and good cognitive development. Fear of self-expression in adolescents at school is significantly reduced due to the absence of manifestations of negative emotions (sadness, resentment, anger) and the absence of external educational motivation to avoid troubles. Attention to internal cognitive and regulatory processes and states increases fear of self-expression in adolescents at school. The absence of negative emotional response to school events and ease in coping with negative emotions help to reduce fear of knowledge testing in adolescents. In contrast, planning a school day increases it. Overall emotional wellbeing reduces fear of a teacher, while satisfaction with relations with classmates increases it.

The academic success of a student is primarily determined by his/her positive emotional state and good cognitive development.

Fear of self-expression in adolescents at school is significantly reduced due to the absence of manifestations of negative emotions (sadness, resentment, anger) and the absence of external educational motivation to avoid troubles. Attention to internal cognitive and regulatory processes and states increases fear of self-expression in adolescents at school.

The absence of negative emotional response to school events and ease in the regulation of negative emotions helps to reduce fear of knowledge checks in adolescents. In contrast, planning of the school day increases it. Overall emotional wellbeing reduces fear of a teacher, while satisfaction with relations with classmates increases it. The strongest predictors of overall school anxiety in adolescents are those which can be combined together, representing two new variables being school wellbeing based on physical wellbeing, self-regulation, and social adaptation, and school wellbeing based on autonomy and personal maturity.

## 7. Limitations

The importance of assessing dynamics of school anxiety and school wellbeing of junior adolescents studying at middle school is conditioned by the need to study external and internal factors, primarily those negatively affected by it, to timely identify and eliminate psychological and behavioral disorders. Continuous analysis of the state of the wellbeing components in schoolchildren (emotional and cognitive) is necessary, as it makes it possible to develop a program for correcting negative consequences and stimulating positive factors affecting wellbeing. The major practical conclusion of the investigation is that school anxiety is largely conditioned by reflexive problems in the sphere of educational skills, the ability for self-regulation, and the self-organization of an adolescent. Despite the high importance of interpersonal relations between adolescents and educational units at various levels, it is obvious that the majority of adolescents do not pose a significant threat to their wellbeing and, accordingly, to their academic adaptation. First of all, the limitation of the study is a small sample. It is also limited by the type of school (general education). The fact that the analyzed data were obtained only from students themselves, and including an issue of success in educational activities, is the limitation. The lack of a standardized tool aimed to assess various aspects of school wellbeing also presents a certain limitation. We attempted to overcome it by creating scales that define different aspects of school life. The results also have limitations associated with their application in practice for improving school wellbeing and reducing school anxiety in junior adolescents studying at secondary schools. It would be important to analyze the school wellbeing in adolescents of different health statuses (with and without health restrictions). The following studies could also compare the school wellbeing in adolescents studying at different types of schools (general school, school with profile, gymnasiums, lyceums).

## Figures and Tables

**Table 1:** Mean values and factor loading of school wellbeing/ill-being characteristics.

Parameters	M	SD	Components			
			1	2	3	4
Desire to become (to be) a good student	3.59	0.73	0.80			
Taking care of school supplies autonomously	3.68	0.78	0.77			
Motivation for achieving success	3.38	0.86	0.75			
Emotionally-positive attitude to lesson material	3.53	0.80	0.72			
Cognitive motivation	3.04	1.01	0.72			
Satisfaction with communication with teachers	3.53	0.76	0.69			
Evaluation of relations with teachers	3.58	0.72	0.62			
Reflection on classwork and mistakes	3.02	1.05	0.60			
Autonomy in doing one’s homework and organization of one’s academic work	3.48	0.67		0.69		
Defending one’s position in a dispute	3.32	0.88		0.67		
Satisfaction with one’s studies	3.20	0.86		0.54		
Autonomy in the academic process	3.34	0.65		0.51		
Self-evaluation of academic success	3.09	0.67		0.48		
Satisfaction with relations with classmates	3.72	0.66			0.77	
Lack of unpleasant physical sensations before school, at school and after school	2.43	1.12			0.62	
Self-regulation of situational emotional disorders	2.98	1.08			0.61	
Well-developed cognitive sphere (attention, memory, comprehension, speech)	3.08	0.90			0.59	
Adaptability self-assessment	3.29	0.85			0.53	
Using techniques and tools that improve well-being, increase confidence in school	3.09	1.08				0.68
Planning the school day	2.53	1.21				0.60
Ability to fight back an offender	3.36	0.87				0.56
Desire to be better in the eyes of others	3.45	0.93				0.52
Dispersion %, total: 61.5			22.6	14.9	14.2	9.8
Alfa			0.89	0.61	0.67	0.65
(Z) Kolmgorov-Smirnov’s test			1.10 *p* > 0.1	0.72 *p* > 0.7	1.05 *p* > 0.08	0.31 *p* > 0.3

Note. The Kaiser–Meyer–Olkin Measure of Sampling Adequacy value is 0.76; Bartlett’s Test of Sphericity Χ^2^ = 704.629, *p* = 0.000.

**Table 2:** Indicators of fit of the confirmatory factor analysis.

Models	Χ^2^	df	*p*	CFI	AGFI	GFI	RMSEA
Single-factor	146.818	114	0.419	0.995	0.834	0.886	0.013
Four-factor	124.781	149	0.926	1.000	0.872	0.909	0.000

**Table 3:** Coefficients of multiple determination and their delta (ΔR^2^/β).

Independent Variables	General Anxiety	Social Stress	Obstacles for Success	Fear of Self-Expression	Fear of Knowledge Check	Fear to Fail One’s Expectations	Low Stress-Resistance	Fear of a Teacher
Lack of negative emotions’ manifestation (sadness, resentment, anger)	0.07/−0.26		0.12/−0.24	0.18/−0.51	0.07/−0.29	0.25/−0.36		0.05/−0.24
Good mood at school	0.04/−0.22	0.03/−0.18	0.05/−0.24					
Satisfaction with relations with classmates		0.07/−0.31						0.06/0.26
Emotionally-positive attitude to lesson material				0.05/0.29				
Well-developed cognitive sphere (attention, memory, comprehension, speech)			0.07/−0.19			0.10/−0.24		
Mental modeling of an answer in class	0.06/0.28							
Autonomy in doing homework and organizing one’s studies		0.03/0.19						
Using methods and tools that improve one’s general state and self-confidence at school		0.32/−0.24		0.06/0.20				
Ability to cope with emotional disorder	0.19/−0.32				0.10/−0.26		0.16/−0.28	0.16/−0.4
Desire to become (be) a good student			0.26/−0.24					
Self-evaluation of academic success			0.03/−0.20					
Planning one’s school day					0.13/0.41			
Autonomy in the academic process						0.04/−0.21		
Defending one’s position in a dispute		0.05/−0.31						
Lack of external academic motivation to avoid trouble		0.03/−0.24		0.05/−0.24		0.05/−0.22		
R^2^	0.36	0.53	0.52	0.34	0.30	0.44	0.16	0.27
F	17.6 **	11.6 **	16.7 **	9.9 **	10.5 **	14.6 **	14.5 **	9.1 *

Legend. Significance value. * *p* < 0.01. ** *p* < 0.05.

**Table 4:** Coefficients of multiple determination and their delta (ΔR^2^/β) for general anxiety.

Independent Variables	β	t	*p*	ΔR^2^
School well-being based on physical well-being, self-regulation and social adaptation	−0.322	−3.18	0.001	0.11
School well-being based on autonomy and personal maturity	−0.314	−3.11	0.001	0.10
	F = 10.06, *p* < 0.001; R^2^ = 0.21			

## Data Availability

Not applicable. The data will be uploaded to https://osf.io.
